# Unusual metachronous lung adenocarcinomas harboring EGFR L858R/T790M mutations: A case report

**DOI:** 10.1111/1759-7714.13618

**Published:** 2020-08-12

**Authors:** Guotian Pei, Shanbo Cao, Yuqing Huang

**Affiliations:** ^1^ Department of Thoracic Surgery, Beijing Haidian Hospital Haidian Section of Peking University Third Hospital Beijing China; ^2^ Acornmed Biotechnology Co., Ltd Beijing China

**Keywords:** Intrapulmonary metastasis, molecular analysis, multiple primary lung cancer

## Abstract

Multiple primary lung cancer (MPLC) is defined as two or more primary lung cancers occurring in the same patient and can be classified as synchronous multiple primary lung cancer (sMPLC) and metachronous multiple primary lung cancer (mMPLC). Due to various clinicopathological characteristics and genetic features, MPLC is increasingly encountered in clinical practice. The distinction between MPLC and intrapulmonary metastasis (IM) is of great importance to clinical treatment and prognosis. However, there are currently no golden diagnostic criteria for MPLC due to tumor heterogeneity. Here, we report the case of a patient with four lung cancers (tumor 1, named T1, in the right middle lobe seven years earlier; tumor 2, named T2, in the left lower lobe; tumor 3 and tumor 4, named T3 and T4, in the left upper lobe) and two tumors (T1 and T2) which shared the mutation in epidermal growth factor receptor (EGFR) L858R/T790M based on targeted multigene sequencing, which indicate that these two tumors might have originated from a common ancestor. However, based on previously published guidelines, these three tumors (T2T4) were diagnosed as mMPLC.

## Graphical abstract

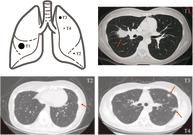

This is the first case of metachronous lung adenocarcinoma harboring EGFR L858R/T790M mutations in a treatment‐naïve non‐small lung cancer patient. Comprehensive clinical and molecular analysis could be helpful in differentiating multiple primary lung cancer from intrapulmonary metastasis.

## Introduction

Multiple primary lung cancer (MPLC) refers to the synchronous or metachronous occurrence of two or more primary lung cancers in a single patient, which can be further divided into synchronous MPLC (sMPLC) and metachronous MPLC (mMPLC) based on the time of occurrence. With the increased availability of high‐resolution computed tomography (CT) and lung cancer screening programs, the reported MPLC incidence rate has been increasing in recent years.^1^

Although the diagnostic criteria for MPLC have been greatly improved, there are currently no golden diagnostic criteria for of MPLC. We herein report the case of a patient with four lung cancers (tumor 1–tumor 4) and two tumors (tumor 1 and tumor 2) which shared the mutation in epidermal growth factor receptor (EGFR) L858R/T790M, and which indicate that these two tumors might have originated from a common ancestor. However, these three tumors (T2–T4) were diagnosed as mMPLC based on the current diagnostic criteria. The following case is presented in accordance with the CARE reporting checklist.

## Case report

**Figure 1:**
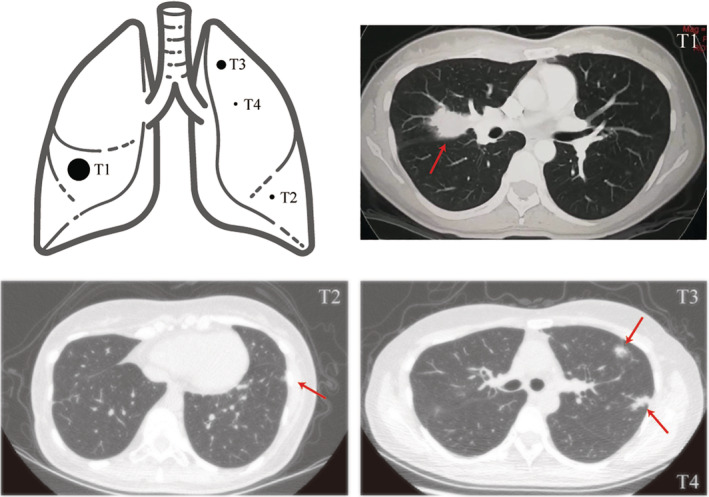
The schematic diagram and chest computed tomography (CT) images of multiple pulmonary nodules in the bilateral lung (arrow).

A 49‐year‐old Chinese woman who was a never smoker was admitted to the Department of Thoracic Surgery of Beijing Haidian Hospital on 13 November 2019 after multiple pulmonary nodules including two nodules in the left upper lobe and one nodule in the left lower lobe had been detected during a health examination (Fig 1). Her physical examination was unremarkable. No distant metastases were found on routine examination. According to the patient, she was diagnosed with stage IIb (pT1N1M0) poorly differentiated lung adenocarcinoma (mixed subtype) in the right middle lobe (tumor 1, named T1) which was treated with right middle lobectomy and followed by four cycles of pemetrexed‐cisplatin chemotherapy (pemetrexed 500 mg/m^2^ day 1, cisplatin 75 mg/m^2^ day 1, for 21 days as a cycle) seven years previously.

**Figure 2:**
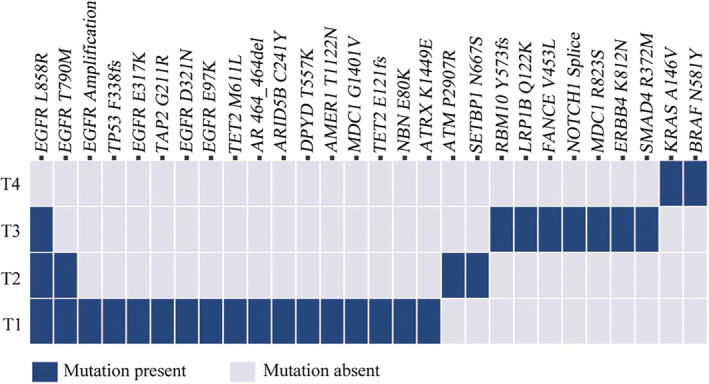
The mutation distributions of the tumor lesions (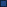) Mutation present, (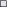) Mutation absent.

She then underwent wedge resections of the left upper lobe and left lower lobe and lymph node dissection by video‐assisted thoracoscopic surgery (VATS). The postoperative pathological subtype of each lesion was acinar predominant adenocarcinoma (tumor 3, named T3) and atypical adenomatous hyperplasias (tumor 4, named T4) in the left upper lesion and minimally invasive adenocarcinoma (tumor 2, named T2) in the left lower lesion. No lymph node metastasis was observed. Next‐generation sequencing (NGS) test for a large panel containing 808 oncogenic genes was used for molecular testing of all available tumor and matched blood samples (Fig 2).

**Figure 3:**
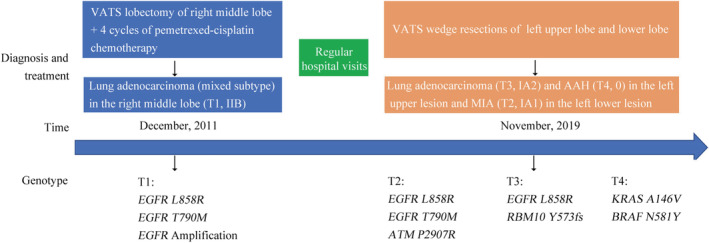
Timeline and genetic analysis of lesions. AAH, atypical adenomatous hyperplasias; MIA, minimally invasive adenocarcinoma.

We found that three lesions (T1, T2 and T3) shared a single mutation in *EGFR* L858R. The lesions T1 and T3 displayed different major histological subtypes and were therefore considered as separate primary lung cancers. No shared mutations were identified between lesion T4 and other lesions, and we concluded that T4 was from an independent clonal origin. Interestingly, the lesions T1 and T2 shared the mutation in *EGFR* L858R/T790M (Fig 2), suggesting that these two lesions might have originated from a common ancestor. However, based on the Martini and Melamed criteria^2^ or the American College of Chest Physicians (ACCP) guidelines,^3^, ^4^, ^5^ these three lesions (T2‐4) were diagnosed as mMPLC (pT1bN0M0, stage IA2). Because the tumors were still at an early stage, the patient did not receive adjuvant therapy but continued to be monitored through regular hospital visits every three months. To date, no recurrence or metastasis was found in this patient (Fig 3).

## Discussion

With advances in imaging technology, MPLC is increasingly encountered in clinical practice. The accurate diagnosis and treatment of MPLC has become an urgent concern.^1^ However, it is difficult to distinguish the second primary lesion and the metastasis. In 1924, Beyreuther described cases of “double primary lung cancer” and introduced the concept of MPLC for the first time.^6^ The clinicopathological criteria for the diagnosis of MPLC was established by Martini and Melamed in 1975^2^ and then revised by Antakli *et al*.^7^ and Colice *et al*.^8^ who extended the required disease‐free interval up to four years. However, this empirical classification did not include molecular analysis, which is now routinely performed for lung adenocarcinoma.^9^ The ACCP guidelines then developed the diagnostic criteria with further clinical evaluations including histological subtypes of adenocarcinoma and molecular genetic characteristics.^3^, ^4^, ^5^ Although the diagnostic criteria have been greatly improved, the diagnosis and classification of MPLC has still not reached consensus amongst the Union for International Cancer Control (UICC), American Joint Committee on Cancer (AJCC), and International Association for the Study of Lung Cancer (IASLC).^5^, ^10^, ^11^

Nowadays, recent studies have revealed that comprehensive molecular analysis are playing important roles in the differentiation of MPLC and IM.^12^, ^13^, ^14^, ^15^ In general, tumors with largely concordant mutations have been considered as clonal in origin (metastases), and those with discordant mutations considered to be primary tumors. However, the same mutations may occur in morphologically different tumors and driver mutations, especially for *EGFR* germline mutation, which can also complicate the interpretation of the clonal relationship of multiple lung adenocarcinoma.^16^, ^17^ Therefore, significant caution is advised when interpreting the same mutations in two tumors.

In this report, our patient was an unusual case with early‐stage metachronous multiple lung cancers and treatment‐naïve *EGFR* L858R/T790M mutations in two tumors seven years after right middle lobectomy. How do we explain this phenomenon? On the one hand, these three tumors (T2–T4) could be diagnosed as mMPLC based on the current diagnostic criteria.^2^, ^3^, ^4^, ^5^ On the other hand, treatment‐naïve NSCLC with *EGFR* T790M mutation is rare, and has only been reported in around 2% of patients with *EGFR*‐mutant lung cancer.^18^ Furthermore, concurrent mutations in *EGFR* L858R/T790M have been reported in around 0.5% of *EGFR* mutation‐positive patients before exposure to EGFR‐tyrosine kinase inhibitors (TKIs).^19^ In this report, T1 and T2 shared the *EGFR* L858R/T790M mutation, suggesting that these two lesions might have originated from a common ancestor. However, despite being provided with all this evidence, we were unsure whether T2 was an IM or not, and it was determined that the results needed to be combined with the patient's prognosis in order to make an accurate judgment. Long‐term follow‐up and survival data would also be critical in substantiating our observations.

This is the first case, to our knowledge, of metachronous lung adenocarcinoma harboring *EGFR* L858R/T790M mutations in a treatment‐naïve non‐small lung cancer (NSCLC) patient. Comprehensive clinical and molecular analysis may be helpful in differentiating MPLC from IM. Due to the ambiguous criteria for the diagnosis of MPLC, more emphasis should be placed on this in future studies.

## Disclosure

Shanbo Cao is an employee of Acornmed Biotechnology Co., Ltd. All other authors have nothing to disclose.
